# Evaluating the safety of intraoperative instillation of intravesical chemotherapy at the time of nephroureterectomy

**DOI:** 10.1186/s12894-015-0039-0

**Published:** 2015-05-28

**Authors:** Michael A. Moriarty, Matthew A. Uhlman, Megan T. Bing, Michael A. O’Donnell, James A. Brown, Chad R. Tracy, Sundeep Deorah, Kenneth G. Nepple, Amit Gupta

**Affiliations:** Department of Urology, University of Iowa, 200 Hawkins Drive, 3RCP, Iowa City, IA 52242 USA

**Keywords:** Carcinoma, Intravesical chemotherapy, Intraoperative mitomycin C, Nephroureterectomy, Urothelial carcinoma

## Abstract

**Background:**

Urothelial carcinoma (UC) is a common cancer affecting many patients in the United States. Nephroureterectomy remains the gold standard for the treatment of high grade upper tract disease or low grade tumors that are not amenable to endoscopic management. Recent reports have shown a decrease in UC recurrence in patients who underwent nephroureterectomy and who had Mitomycin C (MMC) instilled into the bladder at the time of catheter removal. At our institution instillation of intravesical MMC at the time of nephroureterectomy has been common for more than 10 years. Given the recent data, we sought to formally describe our experience with and evaluate the safety of intravesical instillation of cytotoxic chemotherapy at the time of nephroureterectomy.

**Methods:**

We retrospectively reviewed 51 patients who underwent intraoperative intravesical instillation of cytotoxic chemotherapy (MMC (*n* = 48) or adriamycin (*n* = 3)) at the time of nephroureterectomy (2000–2012). The procedure was performed in a similar fashion by 8 different surgeons from the same institution, with drainage of the bladder prior to management of the bladder cuff. Patient characteristics and perioperative data including complications out to 90 days after surgery were collected. Perioperative complications for all patients were graded using the modified Clavien-Dindo classification.

**Results:**

Twenty-four men and 27 women underwent intraoperative intravesical instillation of cytotoxic chemotherapy at the time of nephroureterectomy. Median age at the time of operation was 74 years (range 48–88). Median dwell time was 60 min. Twenty three patients had a total of 45 perioperative complications. The majority (36/45) were Clavien grades I and II. No patients experienced any intraoperative or postoperative complications attributable to MMC or Adriamycin instillation.

**Conclusion:**

Intraoperative intravesical instillation of cytotoxic chemotherapy at the time of nephroureterectomy is safe and feasible. Multicenter trials to study the efficacy of early cytotoxic chemotherapy administration to prevent recurrence of bladder urothelial carcinoma following nephroureterectomy are warranted.

## Background

Urothelial carcinoma of the bladder is the sixth most common cancer affecting patients in the United States [1]. The majority of urothelial carcinomas are found in the bladder, but upper tract disease accounts for approximately 5–10 % of urothelial carcinomas [2, 3]. Nephroureterectomy is the standard treatment for high grade upper tract disease or low grade tumors that are not amenable to endoscopic management [4]. However, the recurrence rate for urothelial carcinoma in the bladder is still high, with reports ranging from 25 % to nearly 70 % [5–11]. While some recurrence may be due to a field effect, it has been hypothesized that recurrence in the bladder may be due to implantation of sloughed neoplastic cells from the upper tract. This hypothesis is supported by several molecular studies showing matching molecular markers and DNA sequence changes in upper tract primaries and subsequent recurrences in the lower tract [12–16].

Strategies have been developed to prevent recurrence of urothelial carcinoma. Early during nephroureterectomy the ureter can be clipped to prevent tumor migration with kidney manipulation. Additionally, intravesical therapy has been utilized [10, 11]. Intravesical instillation of mitomycin C (MMC) after transurethral resection of bladder tumor (TURBT) has been shown to reduce recurrence of urothelial carcinoma in the bladder in several studies and is the current standard of care in the USA and Europe [17, 18]. More recently, 2 randomized studies have looked at the effect of intravesical MMC instillation on urothelial carcinoma recurrence in the bladder following nephroureterectomy. The ODMIT-C trial, a multicenter randomized trial, demonstrated a decrease in bladder recurrence in patients who received a single dose of intravesical MMC at various times postoperatively prior to catheter removal [10]. Another smaller randomized controlled study showed similar decreases in recurrence with a 30-min intravesical instillation of pirarubicin (THP) performed within 48 h after nephroureterectomy [11]. In that study, the 36 patients who received THP and were analyzed for recurrences had a lower risk of bladder recurrence in comparison to the non-treatment group.

A common theme between these studies was that instillation of the cytotoxic agent was performed days to weeks following surgery. This delay in instillation was due to concerns for spillage into the surgical field if given intraoperatively or extravasation of the cytotoxic agent from the bladder if given postoperatively before the bladder has healed. However, it is known that following bladder tumor resection MMC is most effective in preventing tumor implantation if given within 6 h of surgery [19, 20]. Hence, it is possible that intraoperative use of MMC at time of nephroureterectomy may be better than delayed instillation. To our knowledge, due to lack of safety data, no studies to date have examined the efficacy of intravesical instillation of MMC at the time of nephroureterectomy. This safety data would be critically relevant in the design of future prospective studies that evaluate earlier administration of intravesical chemotherapy.

## Methods

The University of Iowa Institutional Review Board approved this study prior to the retrospective identification and reviewing of patient records and has therefore been performed in accordance with the ethical standards laid down in the 1964 Declaration of Helsinki and its later amendments.

After receiving approval from the Institutional Review Board, we retrospectively identified and reviewed the records for 51 patients from 2000 to 2012 who had undergone nephroureterectomy and had received intravesical MMC or Adriamycin. Signed informed consent was obtained on all patients prior to surgery and included consent for instillation of MMC or Adriamycin. Given the retrospective nature of the study, it was determined that no additional informed consent was required to examine data. Adriamycin was utilized when MMC was in short supply nationally, which occurred sporadically from 2008 through 2012. Use of cytotoxic intravesical chemotherapy was based on surgeon preference.

For each patient, a two-way catheter was sterilely inserted into the bladder on the operative field at the beginning of the surgical procedure. After the bladder had been completely drained, MMC (40 mg MMC in 40 ml sterile water) was instilled into the bladder and the catheter was clamped. During times of MMC shortage, three patients received intraoperative intravesical Adriamycin (50 mg in 50 ml saline). The catheter was typically left clamped for one to two hours. Kidney and ureteral dissection were performed prior to bladder incision. Intraoperatively, we always attempted to clip or ligate the ureter distal to the cancer site as soon as possible. After one to two hours of dwell time, the catheter was unclamped and the cytotoxic chemotherapy was allowed to drain passively, well before the bladder was opened for distal ureterectomy. The catheter bag was then disposed as cytotoxic waste. The bladder was occasionally irrigated with saline at surgeon discretion. The technique of distal ureteral dissection was left to the discretion of the surgeon. They included cystotomy with an intravesical bladder cuff, extravesical incision of the ureter with a bladder cuff, distal ureterectomy and extravesical dissection of the ureter with an intramural ureterectomy. The majority of cases (30/51) were performed with an extravesical excision of the ureter with a bladder cuff.

Patient charts were reviewed and demographic and clinical data were carefully reviewed. Intraoperative and postoperative complications up to 90 days after surgery were recorded. All patients were seen at least twice within 90 days post operatively. Complications were graded according to the modified Clavien-Dindo grading system [21, 22], which scores deviations in normal postoperative care based on severity.

## Results

We identified 51 patients who received intraoperative intravesical chemotherapy during the study period. Demographic and clinical information presented in Table 1. The majority of patients (36/51) presented with gross hematuria and were smokers (39/51), with a median smoking history of 20 pack years. Median age at time of nephroureterectomy was 74 years. Twenty-eight of the 51 patients had a history of prior bladder cancer.

**Table 1:** Demographic and clinical characteristics of study patients

Variable		Number
Patient Sex		
	Male	24
	Female	27
Median Age, yrs (range)		74 (48–88)
Smoking status		
	Smoker (Current or Past)	39
	Non-smoker	12
Pathological Stage after Nephroureterectomy		
	Ta	14
	Tis	2
	T1	13
	T2	7
	T3	15
Pathologic Grade after Nephroureterectomy		
	LG	16
	HG	34
	Not Reported	1
Distal Ureter Handling		
	Cystotomy with intravesical bladder cuff	9
	Extravesical incision of ureter with bladder cuff	30
	Distal ureterectomy	9
	Extravesical dissection of ureter with intramural ureterectomy	3

The median dwell time for the cytotoxic intravesical chemotherapy was 60 min (range 45–120 min). Both contained a dye such that any extravesical spillage could be identified at the time of surgery. There were no intraoperative complications related to MMC or Adriamycin instillation. Eight patients had intraoperative complications: ureteral transection prior to ureterectomy (1), incomplete bladder closure (1), bowel injury requiring resection (1), acute blood loss requiring transfusion (3), and iliac vein injury requiring repair (1). Intraoperative spillage of MMC into the surgical field was not experienced in any of the surgeries.

Postoperatively, there were a total of 45 complications in 23 patients (Table 2). Twenty-two patients had 0 complications, 7 had 1 complication, 11 had 2 complications, 4 had 3 complications and 1 had 4 complications. The majority of the complications were Clavien grades 1 or 2 (35/45). Grades 3 and 4 postoperative complications included one occurrence each of watershed cerebral infarct, myocardial infarction, respiratory failure requiring ventilation, foley catheter occlusion with clots requiring irrigation, reoperation due to bleeding, acute renal failure requiring dialysis, SICU transfer due to septic shock and urinoma requiring drain placement. There were no complications resulting in prolonged or chronic disability. Notably, the patient who experienced a bladder leak requiring drain placement had no symptoms or signs of peritonitis. No patients experienced any postoperative complications directly attributable to MMC or Adriamycin instillation such as severe or chronic pelvic pain or chemical peritonitis. The median length of stay was 4.0 days (range 2–21 days).

**Table 2:** Severity of post-operative complications

Total number of complications		45
Complications due to MMC Instillation		0
Complication Severity		
	Clavien Grade	
	I	20
	II	16
	IIIa	2
	IIIb	1
	IV-a	5
	IV-b	1
	V	0

## Discussion

Intravesical MMC has been a standard of care for the treatment of bladder cancer for over 30 years. As an extension of that use, physicians at various centers have also administered it intravesically at the onset of performing radical cystectomy or nephro-ureterectomy and then draining the bladder roughly 60 min later, prior to more formal bladder manipulation. This has been our standard clinical practice for more than 15 years and as such was incorporated into our standard operative consent. It was only later that we realized this practice was not uniformly applied elsewhere and, as such, we obtained appropriate IRB approval to perform this retrospective study.

In this study of 51 patients, intraoperative instillation of intravesical cytotoxic therapy at the time of nephroureterectomy was found to be safe. None of the patients experienced any adverse events directly attributable to MMC instillation either intraoperatively or postoperatively. This study provides safety data in support of a prospective trial designed to assess the efficacy of earlier intravesical chemotherapy in the prevention of bladder tumors after nephroureterectomy. Recent studies have found that without the administration of adjuvant postoperative intravesical therapy, the bladder urothelial carcinoma recurrence rate is approximately 25–70 % [10, 11] at median follow-up of 12–45 months. Two randomized studies have shown that in patients with upper tract urothelial carcinoma, postoperative intravesical instillation of a single dose of a cytotoxic agent such as MMC or THP decreases recurrence of bladder urothelial carcinoma. However, in these studies the intravesical cytotoxic agent was administered 2 days to a few weeks after the nephroureterectomy. The ODMIT-C trial was a multicenter phase III study that randomized 284 patients to MMC and control [10]. MMC was administered a median of 7 days after nephroureterectomy, at the time of catheter removal. At one year, there was a 10 % absolute and 40 % relative decrease in the risk of recurrence in the bladder with instillation of MMC. Ito et al. conducted a randomized phase II trial of single-dose intravesical instillation of THP that was administered within 48 h of surgery [11]. This trial randomized 77 patients and found an absolute decrease of 14.9 % and 25.3 % at one and two years, respectively. Interestingly, in both studies the absolute incidence of bladder tumors in the treatment arm was about 16 %, with a number needed to treat of 9 in the ODMIT trial. For patients with bladder urothelial carcinoma, studies have shown the instillation of MMC reduces recurrence rates when administered within 6 h of surgery, but not when it is instilled greater than 24 h after surgery [23–25]. In fact, one randomized controlled trial demonstrated that pre-TURBT electromotive instillation of MMC was superior to post-TURBT MMC. [26].

Hence, it is entirely plausible that the efficacy of MMC in preventing recurrence of the urothelial carcinoma in the bladder may be higher if MMC is administered during or immediately after nephroureterectomy rather than several days later.

Immediate postoperative instillation of MMC in the bladder after TURBT has been shown to be safe even in the setting of tumor resection and some element of continued bleeding, so it is not surprising that our experience with instillation and immediate drainage was not associated with any adverse effects.

In the ODMIT-C trial, the timing of instillation was delayed due to concern for extravasation of MMC into the pelvis from the bladder [10]. Within the THP monotherapy study group trial, administration of intravesical chemotherapy was within 48 h of surgery [11]. The group did not mention the reason for choosing this particular time for instillation, but did note that the bladder cuff resection was performed in an open fashion so as to assure the wall was tightly sutured. In theory, this timing of administration might have decreased the efficacy of these agents.

Despite evidence from these 2 randomized studies [10], postoperative intravesical instillation of MMC at the time of catheter removal has not become standard practice. The reasons for this lack of dissemination and implementation are not known and are likely multi-factorial. Even after TURBT, when safety and efficacy rates are known to be high, the utilization of a single postoperative dose of mitomycin has been reported to be as low as 38 % [27]. One contributing factor may be that intravesical instillation is not built into the workflow of the typical postoperative course, and in many cases may require the patient to return to the clinic after hospital discharge. When patients return for follow-up and catheter removal, the clinic encounter is likely to be focused on the patient’s symptoms and discussion of the pathology report, prognosis and treatment plan. Additional barriers such as the need for timely coordination with the pharmacy, biohazard precautions and the requirement for specialized nursing may make the administration of MMC less likely. Therefore, it is plausible that by standardizing the administration of MMC intraoperatively and engaging the urologic team directly, it may increase the probability of patients receiving this therapy.

At our institution, a number of physicians (8 over the last 12 years) have instilled cytotoxic chemotherapy in the bladder at the time of nephroureterectomy. To the best of our knowledge, we are the first to report on the safety of this approach. Our experience with 51 patients has been a highly positive one. All patients tolerated the instillation without issue and no intra or postoperative complications related to cytotoxic chemotherapy occurred. A larger prospective study would provide greater reliability on the safety of this approach as well as more reliable data in regard to its efficacy, which theoretically could be better than the previously described randomized studies.

There are number of limitations that should be addressed. Our review included data from 51 patients who underwent nephroureterectomy from 2000 to 2012 and included 28 patients who had a prior history of bladder cancer. As such, time to recurrence of bladder cancer is not meaningful and efficacy cannot be studied in this cohort. Additionally, a retrospective review of complications may underestimate the number of complications that occurred; however, our data are comparable to a number of previous reports and highlight the relatively low but significant percentage of Clavien grades 3 and 4 complications. Second, the handling of the distal ureter was left to the discretion of the surgereon, though the majority of cases (30/51) involved an extravesical excision of the ureter along with a bladder cuff. Next, there are currently no data on the efficacy of intraoperative intravesical cytotoxic chemotherapy for upper tract urothelial carcinoma, though extrapolation from TURBT data may be appropriate. This does, however, highlight an important reminder from our study – the rarity of upper tract urothelial carcinoma and the inherent difficulty in studying a rare disease. The majority of our patients had a prior history of bladder cancer, whereas to study the efficacy of an intravesical agent, a cohort of patients without prior bladder cancer is needed. Thus, given the rarity of the disease, and our findings demonstrating that intraoperative instillation of intravesical cytotoxic chemotherapy is safe, multicenter prospective trials are needed to determine whether this approach is effective in preventing recurrence of urothelial carcinoma in the bladder following nephroureterectomy. This data provides important preliminary safety data in support of such a trial.

## Conclusion

Intraoperative, intravesical instillation of cytotoxic chemotherapy at the time of nephroureterectomy is safe and feasible. Multicenter clinical trials to study the efficacy of this approach to prevent recurrence of bladder urothelial carcinoma are warranted.

## Abbreviations

UC: Urothelial Carcinoma
MMC: Mitomycin C
TURBT: Transurethral resection of bladder tumor
THP: Pirarubicin
